# Brain stimulation treatment for bipolar disorder

**DOI:** 10.1111/bdi.13283

**Published:** 2022-12-21

**Authors:** Julian Mutz

**Affiliations:** ^1^ Social, Genetic and Developmental Psychiatry Centre Institute of Psychiatry, Psychology & Neuroscience, King's College London London UK

**Keywords:** bipolar and related disorders, bipolar depression, bipolar disorder, deep brain stimulation, electroconvulsive therapy, magnetic seizure therapy, mania, transcranial direct current stimulation, transcranial magnetic stimulation, vagus nerve stimulation

## Abstract

**Aims:**

Bipolar disorders are clinically complex, chronic and recurrent disorders. Few treatment options are effective across hypomanic, manic, depressive and mixed states and as continuation or maintenance treatment after initial symptom remission. The aim of this review was to provide an up‐to‐date overview of research on the efficacy, tolerability and cognitive effects of electroconvulsive therapy (ECT), transcranial magnetic stimulation (TMS), transcranial direct current stimulation (tDCS), magnetic seizure therapy (MST), deep brain stimulation (DBS) and vagus nerve stimulation (VNS).

**Methods:**

References included in this review were identified through multiple searches of the Embase, PubMed/MEDLINE and APA PsycINFO electronic databases for articles published from inception until February 2022. Published reviews, meta‐analyses, randomised controlled trials and recent studies were prioritised to provide a comprehensive and up‐to‐date overview of research on brain stimulation in patients with bipolar disorders.

**Results:**

The evidence base for brain stimulation as an add‐on or alternative to pharmacological and psychological treatments in patients with bipolar disorders is limited but rapidly expanding. Brain stimulation treatments represent an opportunity to treat all bipolar disorder states, including cognitive dysfunction during euthymic periods.

**Conclusion:**

Whilst findings to date have been encouraging, larger randomised controlled trials with long‐term follow‐up are needed to clarify important questions regarding treatment efficacy and tolerability, the frequency of treatment‐emergent affective switches and effects on cognitive function.

## 1 INTRODUCTION

Bipolar disorders are characterised by recurrent episodes of mood disturbances and changes in energy level and behaviour, including periods of depression, hypomania or mania, mixed states, subsyndromal symptoms and euthymia. Bipolar disorders rank amongst the most debilitating non‐communicable diseases,^1^ with an estimated lifetime prevalence of 1.06% for bipolar disorder type I and 1.57% for bipolar disorder type II.^2^ The annual costs of managing bipolar disorders in the UK were estimated at £342 million,^3^ with hospital inpatient care representing the largest cost factor.^3^, ^4^ Depressive episodes are more prevalent than (hypo)manic episodes and are associated with a greater disease burden.^5^, ^6^ An estimated 55.2% of patients experience a recurrent episode within 2 years.^7^ Mixed states are less common but are associated with an increased risk of suicide and high levels of comorbidity.^8^ Many individuals with bipolar disorders present with functional impairments,^9^ have an elevated risk of morbidity and mortality,^10^, ^11^ and report comorbid substance use disorders which may negatively affect their long‐term prognosis.^12^ Cognitive impairments are common, also during euthymic periods, and correlate with reduced daily functioning.^13^ The elevated suicide risk,^14^ high prevalence of comorbidities and accelerated biological ageing^15^, ^16^, ^17^ may contribute to the lower life expectancy of individuals with bipolar disorders.^18^ For an overview of the epidemiology, pathophysiology, diagnosis and treatment of bipolar disorders, see Vieta et al. (2018).^19^

Treatments include medications^20^ and psychotherapy.^21^ Manic episodes are treated primarily with pharmacological monotherapy or a combination of antipsychotics with lithium or anticonvulsants.^22^ Treatment with antidepressant medications may be associated with an increased risk of treatment‐emergent affective switches,^23^ highlighting the challenge of treating depressive episodes.^24^ Treatment‐resistant bipolar depression is a major contributor to the overall disease burden.^25^ Due to their clinically complex presentation, mixed states are difficult to treat and often unresponsive to pharmacotherapy.^26^ The use of multiple medications in combination is common in patients with bipolar disorders^27^ and may be associated with adverse effects that contribute to non‐adherence or treatment discontinuation. Undesired effects include weight gain, metabolic dysregulation and sedation.^28^ Cognitive function is critical to daily functioning and an important component of treatment response, given that many individuals with bipolar disorders show cognitive impairment.^29^ However, pharmacological treatments are primarily targeted towards the improvement of mood symptoms, and few treatments improve cognitive dysfunction.^30^ As such, there is a need to improve existing treatments and develop novel therapeutic options that are safe, effective and improve other features of bipolar disorders, including cognitive dysfunction.

There is a growing interest in brain stimulation as an add‐on or alternative to existing treatments in patients with bipolar disorders. Much has been learned about the efficacy of brain stimulation treatments for major depressive episodes,^31^, ^32^, ^33^ mostly in patients who did not respond to medication.^34^ However, it is unclear to what extent this evidence base applies to bipolar depression, and how effective recent treatment protocols are in hypomanic, manic or mixed states. A 2016 review of brain stimulation for treatment‐resistant bipolar disorders concluded that limited data were available to evaluate the efficacy and safety in this patient group, except for electroconvulsive therapy (ECT).^35^ ECT is indicated for bipolar depression, mania and mixed features in several national and international treatment guidelines, however, recommendations regarding other brain stimulation treatments vary.^36^, ^37^, ^38^, ^39^, ^40^, ^41^, ^42^, ^43^

In this review, I summarise the evidence for the most common brain stimulation treatments for bipolar disorders (Panel 1). The review is divided into four main sections: (i) electroconvulsive therapy, (ii) transcranial magnetic stimulation, (iii) transcranial direct current stimulation and (iv) magnetic seizure therapy, deep brain stimulation and vagus nerve stimulation. Each section includes a brief introduction, a review of research on treatment efficacy, effects on cognition and tolerability and a brief conclusion. Where possible, the research on treatment efficacy is presented separately for bipolar depression, (hypo)mania, mixed states, maintenance and euthymia. The review ends with a concluding remark, key challenges for the field and directions for future research.

## 2 SEARCH STRATEGY

References included in this review were identified through multiple searches of the Embase, PubMed/MEDLINE and APA PsycINFO electronic databases for articles published from inception until February 2022, using combinations of the following search terms: ‘bipolar’, ‘bipolar disorder’, ‘transcranial direct current stimulation’, ‘tDCS’, ‘transcranial magnetic stimulation’, ‘TMS’, ‘theta burst stimulation’, ‘TBS’, ‘electroconvulsive therapy’, ‘ECT’, ‘magnetic seizure therapy’, ‘MST’, ‘vagus nerve stimulation’, ‘VNS’, ‘deep brain stimulation’ and ‘DBS’. Additional references were identified through backward and forward citation searching in Google Scholar. Due to the breadth of relevant studies, published reviews, meta‐analyses, randomised controlled trials and recent studies were prioritised to provide a comprehensive and up‐to‐date overview of research on brain stimulation in patients with bipolar disorders.

PANEL 1 **Overview of the brain stimulation treatments included in this review**

**Electroconvulsive therapy (ECT)**

Electroconvulsive therapy uses an electric current applied to the head which induces a generalised seizure. The procedure is repeated 2–3 times per week. The patient is sedated with general anaesthesia and given a muscle relaxant to prevent movement during the procedure.

**Transcranial magnetic stimulation (TMS)**

Transcranial magnetic stimulation uses a magnetic field to depolarise superficial neurons and cause action potentials in the brain. TMS can be targeted to a specific site in the brain and does not require the administration of an anaesthetic agent or a muscle relaxant.

**Transcranial direct current stimulation (tDCS)**

Transcranial direct current stimulation changes cortical tissue excitability through the application of a weak (typically 0.5–2 mA) direct current via scalp electrodes overlying targeted cortical areas.

**Magnetic seizure therapy (MST)**

Magnetic seizure therapy uses high‐frequency magnetic pulses to stimulate a specific target in the brain and to induce a seizure. The patient is sedated with general anaesthesia and given a muscle relaxant to prevent movement.

**Deep brain stimulation (DBS)**

Deep brain stimulation involves the surgical implantation of electrodes into the brain. The electrodes are connected to a neurostimulator that is implanted underneath the skin just above the chest. Electrical stimulation is delivered to specific brain regions.

**Vagus nerve stimulation (VNS)**

Vagus nerve stimulation involves the electrical stimulation of the afferent projections of the vagus nerve to the brain. During surgery, electrodes are wrapped around the left vagus nerve and connected to a pulse generator that is implanted underneath the skin near the upper chest.

## 3 ELECTROCONVULSIVE THERAPY

**FIGURE 1:**
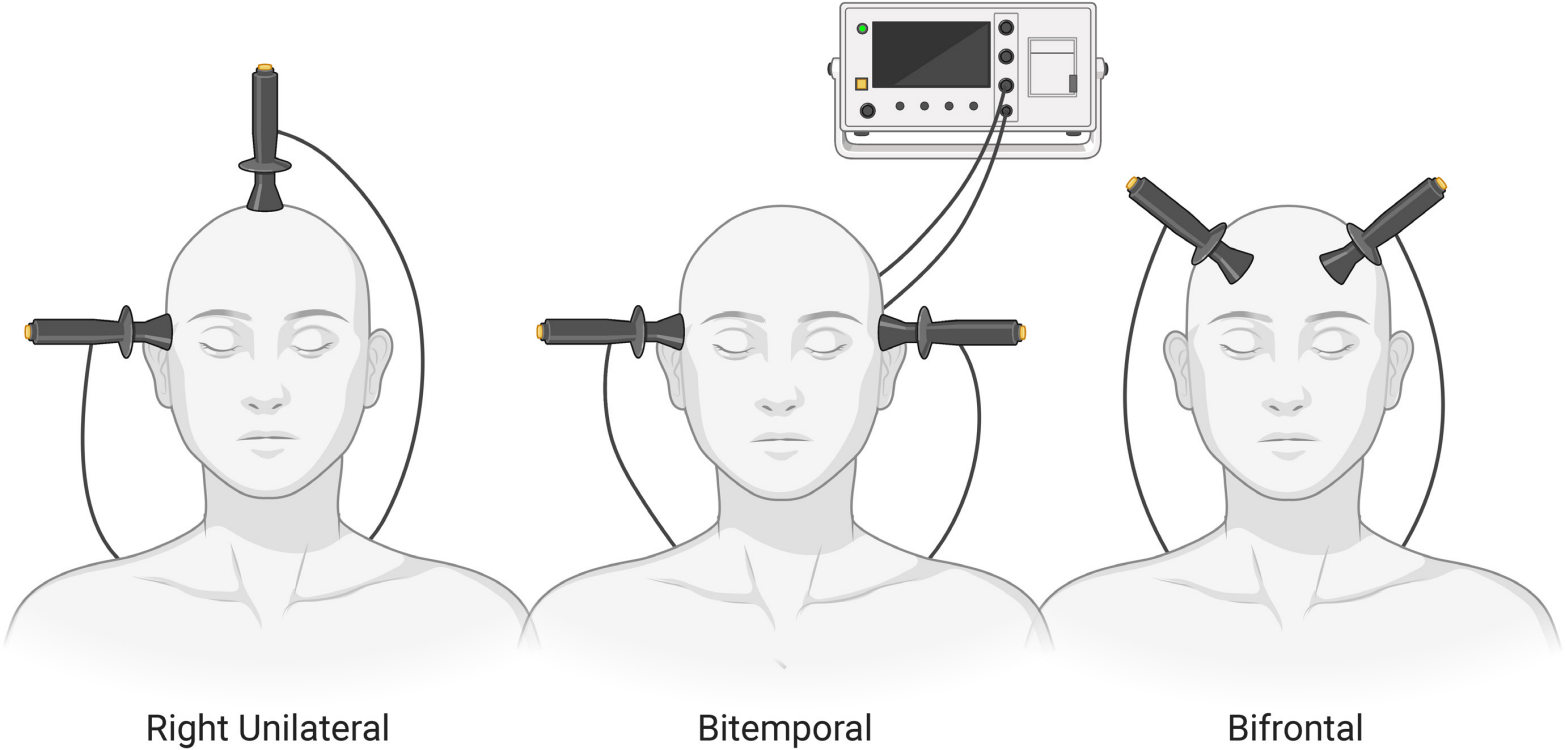
Overview of the main ECT electrode placements. Right unilateral electrode placement (left), bitemporal electrode placement (middle) and bifrontal electrode placement (right).

Electroconvulsive therapy (ECT) was introduced in 1938. Its technology and treatment delivery have since seen major improvements. Treatment protocols differ primarily in electrode placement (bitemporal, right unilateral or bifrontal), electrical dosage (low, moderate or high) and pulse width (brief or ultra‐brief) (Figure 1). For an overview of the history of ECT, its clinical use, mechanisms of action and technical parameters, see McDonald et al. (2017).^44^

### 3.1 Efficacy

#### 3.1.1 Bipolar depression

Multiple studies suggest that ECT is an effective treatment for bipolar depression. A meta‐analysis of six prospective and retrospective studies found remission rates of 50.9% (*n* = 402/790) in patients with major depressive disorder (MDD) and 53.2% (*n* = 168/316) in patients with bipolar depression, suggesting similar effectiveness in both groups.^45^ Notably, most bipolar disorder patients were more severely ill. Another review of 19 studies found that response rates were slightly higher in patients with bipolar depression (77.1%, *n* = 437/567) than in patients with MDD (74.2%, *n* = 1246/1680).^46^ Bipolar disorder patients required fewer sessions to respond to treatment (SMD = −0.23, 95% CI −0.44 to ‐0.02, *p* = 0.03). Consistent with the earlier meta‐analysis,^45^ there was no evidence of group differences in remission rates (OR = 0.91, 95% CI 0.65–1.26, *p* = 0.56). Randomised controlled trials (RCTs) of ECT in bipolar depression are scarce, and there are no sham‐controlled trials. A trial comparing ECT to algorithm‐based pharmacotherapy in 73 patients found a higher response rate in patients treated with ECT, but similar remission rates in both groups.^47^ Data from a retrospective study suggested that ECT had better anti‐suicidal effects than pharmacotherapy in patients with MDD or bipolar depression.^48^ However, there was no evidence of superior anti‐suicidal effects in patients with mania or mixed states.^48^ Research on ECT in older adults with bipolar depression is limited and the largest retrospective study included 34 patients aged 65 years or older.^49^ In this study, patients had a reduced illness severity and better cognitive function after treatment and experienced no serious adverse events.

#### 3.1.2 Mania

Two important reviews have shown that ECT is an effective treatment for hypomanic or manic episodes. The first review summarised retrospective and prospective studies conducted before 1992, concluding that ECT was associated with clinical improvement or remission in 80% of 589 manic patients.^50^ A recently updated review included data from 115 publications on the efficacy and safety of ECT in patients with (hypo)mania or mixed states.^51^ Of the seven RCTs to date (*n* = 309), two trials compared ECT to pharmacotherapy, one trial compared ECT to sham ECT (both combined with chlorpromazine) and four trials compared different ECT protocols. Most of these trials supported the efficacy of ECT in mania. The only sham‐controlled RCT included 15 patients per group and found a higher recovery rate and an accelerated treatment response with ECT.^52^ Observational studies also suggest that ECT is an effective treatment for mania.^51^ However, most patients received concomitant medications, sample sizes were often limited and few studies have examined the relative efficacy of different treatment parameters. A review by Loo et al. (2011) concluded that ECT was likely more effective than pharmacological treatments for mania.^53^ A recent meta‐analysis suggested that combining ECT with pharmacotherapy was more efficacious than medications alone.^54^

#### 3.1.3 Mixed states

ECT has been used to treat patients with mixed states since at least 1984, with a 70% response rate in 19 patients.^55^ However, its effectiveness remains underresearched.^56^ No RCTs have compared ECT to pharmacotherapy or sham ECT in this patient group.^53^, ^57^ A 2008 review identified three studies that included 58 patients with mixed states who received ECT.^58^ One study that included 41 patients with mixed states and 23 patients with bipolar depression observed improvements in both groups, with a slightly greater improvement in mixed states.^59^ Another study observed similar effectiveness in bipolar depression (*n* = 38), mania (*n* = 5) and mixed states (*n* = 10).^60^ Notably, the 10 patients with mixed states required more treatment sessions and a longer hospital stay, suggesting that mixed states are more difficult to treat.^60^ A case series of seven patients with mixed states suggested that all patients remitted.^61^ Another review of seven observational studies concluded that there was preliminary evidence for the effectiveness of ECT in patients with mixed states.^57^ The largest study to date included 197 patients with pharmacotherapy‐resistant mixed states, 82 (41.6%) of whom responded and 60 (30.5%) of whom remitted after on average 7.48 treatment sessions.^62^

#### 3.1.4 Maintenance

Maintaining symptom remission is a clinical challenge. No RCTs have examined continuation or maintenance ECT to prevent relapse or recurrence in bipolar disorders. Data from observational studies are limited but provide encouraging results.^63^, ^64^ A retrospective study of 22 patients observed fewer full, but not total, hospitalisation days compared to the period prior to continuation and maintenance ECT.^65^ The authors also summarised data from prior observational studies of continuation and maintenance ECT that included at least some patients with bipolar disorders.^65^ Another review concluded that continuation and maintenance ECT was effective in mood disorders but insufficiently studied and likely underused.^66^ Of note, the data on bipolar disorders were limited to case studies and small observational studies that also included patients with MDD.

### 3.2 Cognition

Adverse cognitive effects, especially memory impairment, remain a concern with ECT. A meta‐analysis of 84 studies that included 2981 patients with MDD found that most adverse cognitive effects were limited to the first 3 days after treatment.^67^ Data on the cognitive effects of ECT in patients with bipolar disorders are limited. Findings from a RCT suggested no differences in cognitive function between patients with bipolar depression who received right unilateral brief‐pulse ECT or pharmacotherapy after 6 months,^68^ although autobiographical memory was worse in the ECT group after the acute treatment.^69^ In patients with mania, preliminary evidence suggests that cognitive outcomes are better after bifrontal ECT than after bitemporal ECT.^51^

### 3.3 Tolerability

Besides cognitive effects, the most common adverse effects include confusion, headaches, nausea and muscle pain.^70^ Treatment‐emergent affective switches occur in 6%–38.6% of patients and may be more common for bitemporal ECT than right unilateral ECT; lithium treatment may lower their risk of occurrence.^51^ However, a systematic review of these data is lacking. For an overview of potential interactions between ECT and pharmacological treatments, see Loo et al. (2011).^53^ Preliminary evidence suggests that patients with bipolar depression had a decreased risk of adverse events with ECT, relative to patients with MDD.^71^ Across clinical indications, ECT‐related mortality was estimated at 2.1 per 100,000 treatment sessions.^72^

### 3.4 Conclusion

ECT has received less attention as a treatment for bipolar disorders than for MDD, for which it is recommended by the National Institute of Clinical Excellence in the UK.^73^ Few RCTs have been conducted in bipolar depression or mania, and none in patients with mixed states or for continuation and maintenance treatment. Results from most RCTs and observational studies have been encouraging. An advantage of ECT is that it may be effective across bipolar disorder states.^74^ Further studies of protocols that aim to reduce adverse cognitive effects (e.g., ultra‐brief pulse), studies in patients with mixed states and studies of continuation and maintenance ECT are needed.^53^

## 4 TRANSCRANIAL MAGNETIC STIMULATION

**FIGURE 2:**
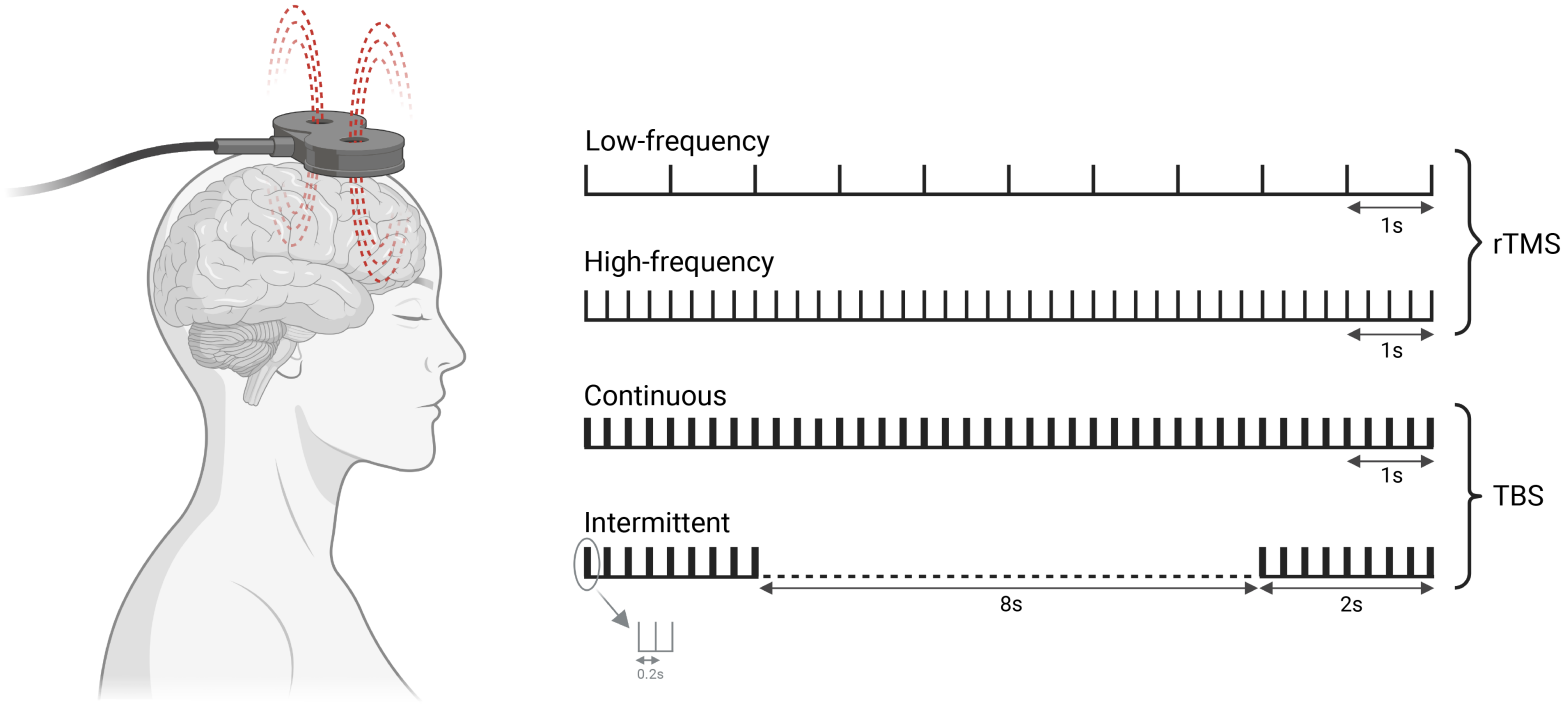
Standard repetitive transcranial magnetic stimulation (rTMS) protocols involve identical stimuli spaced by an identical inter‐stimulus interval. Effects depend on stimulation frequency: low‐frequency rTMS (LF rTMS ≤1 Hz) depresses cortical excitability, whereas high‐frequency rTMS (HF rTMS >5 Hz) increases cortical excitability. Theta burst stimulation (TBS) involves bursts of high‐frequency stimulation (3 pulses at 50 Hz) repeated with an inter‐stimulus interval of 200 ms (5 Hz). In an intermittent TBS (iTBS) protocol, bursts are delivered for 2 s, then repeated every 10 s (2 s of TBS followed by a pause of 8 s). In a continuous TBS protocol (cTBS), bursts are repeated for 40 s without any pause. Adapted from Klomjai et al. (2015).^163^ [Correction added on January 13, 2023, after first online publication: Figure 2 has been updated to correct the order of the labels "High‐frequency" and "Low‐frequency"].

Transcranial magnetic stimulation (TMS) alters neural activity in relatively focal brain regions. Repetitive transcranial magnetic stimulation (rTMS) involves delivering repeated pulses for prolonged neuromodulation. Treatment varies in pulse frequency (low or high) and intensity relative to the motor threshold, and can be targeted unilaterally or bilaterally. High‐frequency rTMS of the left dorsolateral prefrontal cortex (DLPFC) is the most common protocol (Figure 2). More recent approaches include accelerated rTMS, priming TMS, deep TMS (dTMS), synchronised TMS and theta burst stimulation.^32^

### 4.1 Efficacy

#### 4.1.1 Bipolar depression

The first two studies that examined rTMS in mixed samples of patients with bipolar depression or MDD provided preliminary evidence of the efficacy of high‐frequency rTMS of the left DLPFC^75^ and low‐frequency rTMS of the right DLPFC.^76^ A meta‐analysis of data from 19 RCTs included 181 patients with bipolar depression.^77^ Combining data from various TMS protocols, the response rate of 44.3% (*n* = 47/106) was higher than the 25.3% (*n* = 19/75) response rate in the sham control. The most recent meta‐analysis included 274 patients with bipolar depression, 145 of whom received active treatment.^78^ Although rTMS was associated with higher response rates than sham treatment (OR = 2.72, 95% CI 1.44–5.14), subgroup analyses suggested that only high‐frequency rTMS of the left DLPFC was associated with increased response rates. The relative efficacy of different protocols requires further study. A study of 30 patients with bipolar depression observed a higher response rate and similar remission rates in patients treated with bilateral rTMS compared to unilateral stimulation.^79^ Notably, neither of the meta‐analyses examined data on treatment remission, changes in symptom severity scores or global clinical impression.

Few sham‐controlled RCTs have been conducted exclusively in patients with bipolar depression. One trial found that rTMS was efficacious after 2 weeks of treatment, but not after 4 weeks.^80^ A feasibility study of 23 patients, two of whom were in a mixed state, did not find that high‐frequency rTMS of the left DLPFC was more efficacious than sham.^81^ Similarly, a trial of 49 patients that examined sequential bilateral rTMS did not find evidence of efficacy relative to sham.^82^ The first sham‐controlled RCT of dTMS included 50 patients and found that active treatment was superior to sham after 4 weeks of treatment, but not at the 8‐week follow‐up.^83^ Data from open‐label studies of dTMS suggested a significant decrease in Hamilton Depression Rating Scale (HDRS) scores in 19 patients,^84^ and an 80% (*n* = 15/20) response rate in patients treated with bilateral dTMS.^85^

Theta burst stimulation has a great clinical appeal due to its reduced delivery time (<5 min compared to 37.5 min for rTMS). A study of twice daily intermittent theta burst stimulation (iTBS) did not support its efficacy compared to sham.^86^ Similarly, a study of 12 patients with bipolar depression did not find evidence that iTBS was more efficacious than sham.^87^ A recent RCT of iTBS also did not find evidence of efficacy over a 4‐week treatment period.^88^ In this study, 18 patients were randomised to iTBS and 19 to sham control. There were three responders and remitters in each treatment group after the initial course of treatment, and five (of 21 initial non‐responders) during an additional 4 weeks of open‐label treatment. Due to the low response rates, the study was terminated early. Although a TMS device was granted ‘breakthrough designation’ for bipolar depression by the FDA,^89^ additional studies are needed to investigate its efficacy in bipolar depression.^90^

#### 4.1.2 Mania

Multiple reviews explored the efficacy of TMS across bipolar disorder states, concluding that TMS was a promising but understudied treatment.^91^, ^92^, ^93^ Five studies examined the efficacy of rTMS in patients with mania. These studies enrolled 93 patients and provided somewhat inconsistent results, highlighting the need for further research. Most rTMS studies in mania targeted the right DLPFC, following the protocol first used by Grisaru et al. (1998) who found that the improvement in manic symptoms was greater following stimulation of the right than of the left DLPFC.^94^ However, the first sham‐controlled trial in mania found no evidence of efficacy.^95^ Indeed, only one sham‐controlled RCT of rTMS provided evidence of efficacy in mania.^96^ Notably, this was also the largest sham‐controlled RCT.

#### 4.1.3 Mixed states

An open‐label study of low‐frequency stimulation of the right DLPFC included 40 patients with mixed states who received 3 weeks of augmentation treatment, after being treated with a mood stabiliser for 4 weeks.^97^ This study found that rTMS improved both depressive and manic symptoms. The HDRS suggested a 46.6% response rate and the Young Mania Rating Scale had a 15% response rate, and several patients also remitted.^97^ A case report suggested that a patient who did not respond to ECT was successfully treated with high‐frequency rTMS of the left DLPFC during a mixed state.^98^ However, a recent RCT in bipolar disorder and MDD patients with mixed states did not find that bilateral TBS had superior efficacy compared to sham.^99^

#### 4.1.4 Maintenance

A small open‐label study of weekly maintenance rTMS found that three patients successfully completed 1 year of follow‐up whilst four patients had multiple relapses, suggesting that maintenance rTMS was only effective in some patients.^100^ Preliminary evidence also suggests that maintenance dTMS benefitted some patients with bipolar disorders.^101^

### 4.2 Cognition

A review of RCTs found no evidence of differences in cognition between TMS and sham in bipolar depression (two studies) and preliminary evidence that cognitive function improved with TMS during euthymic periods (one study).^102^ The first study to evaluate the cognitive effects of dTMS in bipolar disorders included the per‐protocol subset of the Tavares et al. (2017) RCT.^83^ In this study, 20 patients received dTMS and 23 patients received sham treatment.^103^ All cognitive outcomes improved regardless of the treatment group, suggesting that dTMS was neither associated with adverse nor beneficial cognitive effects. A pilot RCT comparing rTMS (*n* = 16) to sham treatment plus stable pharmacotherapy (*n* = 20) during a non‐acute state found significant improvement in verbal learning in the rTMS group, but no group differences in other cognitive measures.^30^ The largest RCT of rTMS in euthymic patients (*n* = 52) found improved cognitive function across multiple measures relative to sham treatment.^104^

### 4.3 Tolerability

The most common adverse effects are headaches, fatigue, scalp discomfort and pain at the stimulation site.^93^ The risk of TMS‐induced seizures is very low, occurring in less than 1 per 60,000 sessions in patients treated within guidelines and without known risk factors.^105^ To date, no seizures have been reported in patients with bipolar disorders. A meta‐analysis found the rate of treatment‐emergent affective switches following rTMS to be low (0.9%), comparable to sham treatment (1.3%).^77^ Recent reviews identified four cases of (hypo)manic switches.^91^, ^93^ There have also been reports of treatment‐emergent affective switches for iTBS.^88^, ^99^, ^106^

### 4.4 Conclusion

There is a growing evidence base for TMS to treat bipolar depression. Further identification of target brain circuits and nodes is needed to develop these treatments in patients with bipolar disorders.^89^ Few studies support the efficacy of TMS in hypomania, mania or mixed states. Most TMS studies in bipolar disorders examined rTMS as an add‐on to pharmacotherapy, and few studies examined more recent protocols. More research is needed to identify optimal treatment parameters and patient characteristics for treatment stratification. Finally, predictors of treatment‐emergent affective switching remain to be explored as these were not limited to patients treated with antidepressant medication.

## 5 TRANSCRANIAL DIRECT CURRENT STIMULATION

Transcranial direct current stimulation (tDCS) involves delivering a low‐amplitude electrical current through scalp electrodes to modulate cortical excitability of superficial brain regions. Anodal stimulation causes depolarisation and increases neural excitability, whereas cathodal stimulation causes hyperpolarisation and decreases cortical excitability. For an overview of transcranial electrical stimulation and its clinical applications, see Cho et al. (2022).^107^

### 5.1 Efficacy

#### 5.1.1 Bipolar depression

**FIGURE 3:**
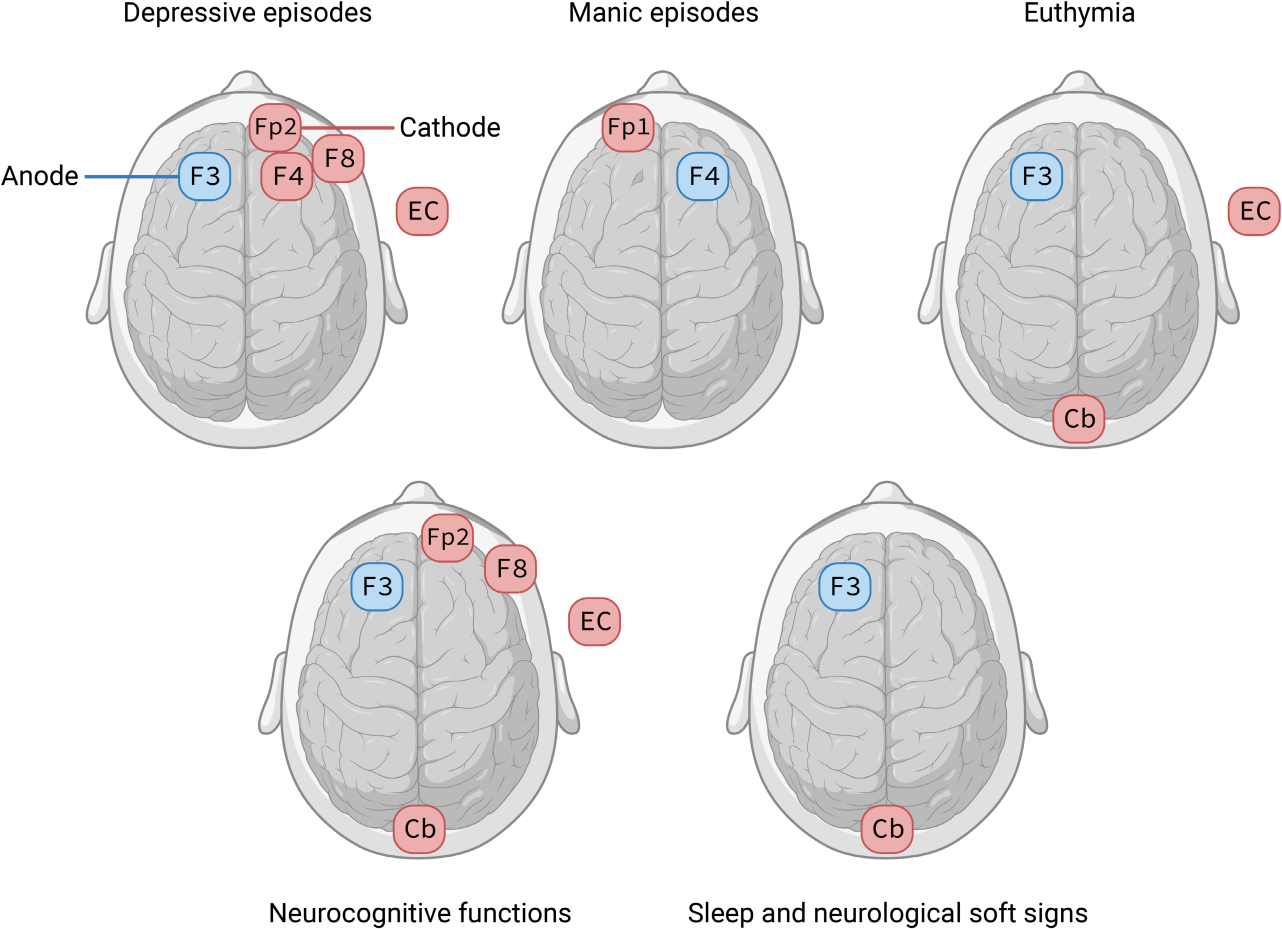
Transcranial direct current stimulation (tDCS) electrode placements used in patients with bipolar disorders: (1) depressive symptoms during depressive episodes, (2) manic symptoms during manic episodes, (3) symptoms during euthymia, (4) neurocognitive functions during depressive episodes or euthymia, (5) sleep and neurological soft signs during euthymia. Blue: anode; red: cathode. Cb, cerebellum; EC, extra‐cephalic (right upper arm); F3, left dorsolateral prefrontal cortex; F4, right dorsolateral prefrontal cortex; F8, right lateral orbit cortex; Fp1, left superior orbital cortex; Fp2, right superior orbital cortex; Electrode placements from Dondé et al. (2018).^116^

Certain treatment guidelines support the use of tDCS in patients with MDD.^108^, ^109^ The first tDCS study that included patients with MDD and bipolar depression observed a decrease in depressive symptoms after five treatment days in both groups.^110^ The first meta‐analysis of tDCS in bipolar depression found that tDCS moderately improved depressive symptoms (SMD = 0.71, 95% CI 0.25–1.18).^111^ However, this analysis included only 46 patients and tDCS was delivered as an add‐on to mood stabilisers. Preliminary evidence in patients with bipolar disorder type I suggested that tDCS combined with pharmacotherapy was associated with a greater depressive symptom reduction than pharmacological monotherapy.^112^ A recent open‐label trial that included 58 patients with MDD and 22 patients with bipolar depression found that tDCS was associated with similar response rates in both groups.^113^ This finding is consistent with an individual patient data meta‐analysis that included 109 (19.1%) patients with bipolar disorders and found no evidence that bipolar disorder status predicted treatment response relative to MDD (OR = 2.28, 95% CI 0.72–7.22).^114^ However, a previous individual patient data meta‐analysis found that bipolar depression predicted increased tDCS response.^115^ A review that examined tDCS as a treatment across bipolar disorder states (Figure 3) included 10 studies (*n* = 76) that found tDCS to be moderately effective in treating depressive symptoms.^116^ The only sham‐controlled RCT exclusively in patients with bipolar depression suggested that tDCS was more efficacious in reducing depressive symptoms than sham treatment.^117^ However, one of the largest tDCS trials to date that included patients with MDD and bipolar depression did not find evidence of an improvement in depression severity after tDCS compared to sham treatment in either patient group.^118^ This discrepancy could be explained by the sham control that was used in the latter trial, which involved the application of a low level of electrical stimulation that could have had a biological effect.

#### 5.1.2 Mania

Data on tDCS in mania are scarce. A case report found that tDCS as an add‐on to pharmacotherapy may improve manic symptoms.^119^ Preliminary evidence also suggested changes in manic symptoms in patients with bipolar depression.^117^, ^120^ However, more research is needed to assess the potential efficacy of tDCS in (hypo)manic patients.

#### 5.1.3 Mixed states

No research has yet examined the efficacy of tDCS in bipolar disorder mixed states.

#### 5.1.4 Maintenance

Data on continuation or maintenance tDCS are scarce. A recent meta‐analysis of patients with depressive episodes, some of whom had bipolar depression, concluded that maintenance tDCS may further improve treatment response.^121^ However, further studies are needed to evaluate the effectiveness of continuation or maintenance tDCS in bipolar disorders.

#### 5.1.5 Euthymia

At least four studies have examined the effects of tDCS on cognition in bipolar disorder patients during euthymic periods.^122^, ^123^, ^124^, ^125^ A study of 15 patients did not observe changes in working memory or sustained attention performance after a single tDCS session.^122^ Although findings from observational studies have been mixed,^124^, ^125^ a RCT found that 3 weeks of prefronto‐cerebellar tDCS (*n* = 21) led to an improvement in executive function and visuospatial memory relative to sham treatment (*n* = 21).^123^ Preliminary evidence from 25 euthymic patients also suggested that tDCS improved neurological function and sleep quality.^126^ These findings are clinically interesting because many patients with bipolar disorder experience sleep disturbance after mood symptom remission.^127^

### 5.2 Cognition

An open‐label study found similar improvements in processing speed, selective attention and executive function in patients with bipolar disorders and MDD after ten sessions of tDCS over five days.^113^ However, a sham‐controlled RCT (*n* = 59) did not provide evidence of post‐treatment group differences in five cognitive domains, suggesting that tDCS neither induced adverse cognitive effects nor improved cognitive function.^128^ Consistent with these findings, data from a meta‐analysis of patients with major depressive episodes found no evidence of cognitive effects that were independent of changes in depressive symptoms.^129^ However, the authors noted the limited data on patients with bipolar disorder. Finally, data from a large RCT suggested that tDCS improved some cognitive functions in patients with bipolar depression.^130^

### 5.3 Tolerability

tDCS is generally well tolerated^131^ and associated with few adverse events.^132^ In patients with bipolar disorder, there have been no reports of major adverse events.^133^ The most common undesired effects of tDCS are mild and transient and include tingling at the skin underneath the electrodes, dizziness and sleepiness. However, no quantitative summary of adverse effects is available,^116^ likely due to insufficient reporting in most studies.^131^ Six cases of treatment‐emergent affective switches (out of 113 patients) were described in the Dondé et al. (2018) review^116^ and one patient included in a recent open‐label study also experienced a new hypomanic episode.^113^ To my knowledge, there have been no reports of rapid cycling or increased risk of suicide associated with tDCS.^111^

### 5.4 Conclusion

Most tDCS studies in patients with bipolar disorders were limited by their open‐label study design and/or small sample size. Only one sham‐controlled trial examined tDCS exclusively in bipolar disorder patients. Although some of the initial results have been encouraging, more research on tDCS in patients with bipolar depression is needed. There are almost no data on patients with hypomanic or manic episodes or mixed states, highlighting the urgent need for studies in these groups. Continuation and maintenance tDCS also remain understudied. Alternative protocols, including the use of multiple electrodes (HD‐tDCS), transcranial alternating current stimulation (tACS) or transcranial random noise stimulation (tRNS), should be explored in bipolar disorders. A key advantage of tDCS and related protocols is the greater portability, which could enable home‐based treatment delivery. Finally, treatment protocols could be stratified by patient characteristics or adjusted at the individual level using machine learning algorithms aimed at optimising treatment parameters.^134^

## 6 OTHER TREATMENT PROTOCOLS

Brain stimulation treatments that have been studied in fewer patients include magnetic seizure therapy, deep brain stimulation and vagus nerve stimulation.

### 6.1 Magnetic seizure therapy

Magnetic seizure therapy (MST) involves the induction of a seizure using magnetic stimulation delivered at a high frequency (typically 100 Hz) under general anaesthesia. The magnetic stimulation avoids impedance of the scalp and skull, does not directly target the temporal lobe regions and causes a shorter seizure than ECT. As such, MST was hypothesised to minimise adverse cognitive effects relative to ECT whilst maintaining its effectiveness.^135^

#### 6.1.1 Efficacy

The evidence base for MST in bipolar disorders is very limited. A 2015 review identified only five patients with bipolar disorders who had received MST.^136^ Two studies provided preliminary evidence that MST might alleviate depressive symptoms. Kayser et al. (2011) observed similar levels of efficacy in patients treated with MST or low‐dose right unilateral ECT.^137^ Kayser et al. (2013) observed faster recovery and reorientation times after MST compared to ECT.^138^ The first open‐label trial exclusively in bipolar depression included treatment‐resistant patients who received up to 24 treatment sessions. The authors observed a reduction in mean HDRS scores from 28.08 (SD = 4.41) at baseline to 17.77 (SD = 8.48) at follow‐up, a 38.5% response rate and a 23.1% remission rate in 26 patients who received at least eight treatments.^139^ In a separate report, the authors showed that MST was associated with reduced suicidal ideation.^140^ 57.1% (*n* = 4/7) of the initial responders maintaining response or remission during a six‐month continuation treatment phase.^141^ To date, there are no published data on the efficacy of MST to treat other bipolar disorder states.

#### 6.1.2 Cognition

Although data on the cognitive effects of MST are scarce,^142^ a previous review did not suggest adverse cognitive effects.^136^ The only study of MST exclusively in patients with bipolar depression observed minimal cognitive effects of MST across 24 tests.^139^

#### 6.1.3 Tolerability

Preliminary evidence suggests that patients with bipolar depression or MDD who received MST reported fewer adverse effects, including less confusion and faster reorientation time, compared to ECT (2–8 min and 18–26 min, respectively).^136^ Although cases of treatment‐emergent mania have been reported in patients with MDD,^143^ there has been only one report of a patient with bipolar depression who received MST and experienced a hypomanic episode several days after the study completion.^139^

### 6.2 Deep brain stimulation

Deep brain stimulation (DBS) involves the surgical implantation of electrodes into the brain which are connected to a neurostimulator that is implanted near the chest. Electrical stimulation is continuously delivered and can be titrated. DBS is primarily used in treatment‐resistant Parkinson's disease and movement disorders but has also been studied in treatment‐resistant obsessive‐compulsive disorder^144^ and treatment‐resistant MDD.^145^

#### 6.2.1 Efficacy

Few studies have examined DBS in patients with bipolar disorders.^146^ The largest sample to date included seven patients with bipolar disorder type II who had a similar decrease in depressive symptoms as ten patients with MDD.^147^ Another case report suggested that a patient with treatment‐resistant bipolar depression was successfully treated with DBS over the course of nine months.^148^ To date, there are no published data on the efficacy of DBS to treat other bipolar disorder states.

#### 6.2.2 Cognition

No studies have examined the cognitive effects of DBS in patients with bipolar disorders.

#### 6.2.3 Tolerability

The most common adverse effects in patients with MDD included pain around the incision, wound infection, headaches and agitation.^149^ Due to the paucity of data on patients with bipolar disorders, there are no published systematic investigations of adverse effects. There have been some reports of treatment‐emergent (hypo)manic symptoms in patients with Parkinson's disease, MDD or obsessive‐compulsive disorder.^150^, ^151^, ^152^ A review of DBS in bipolar disorders^146^ identified one patient who experienced two episodes of hypomania during treatment that were resolved with adjustment of treatment parameters.^153^ No treatment‐emergent affective switches were observed in the largest study to date.^147^

### 6.3 Vagus nerve stimulation

Vagus nerve stimulation (VNS) involves the electrical stimulation of the afferent projections of the vagus nerve to the brain. During surgery, electrodes are wrapped around the left vagus nerve and connected to a pulse generator that is implanted near the upper chest. The stimulation is less focal than DBS but can also be titrated. VNS was first developed for treatment‐resistant epilepsy but has since been approved for treatment‐resistant MDD.

#### 6.3.1 Efficacy

A 5‐year follow‐up study of almost 500 patients with treatment‐resistant MDD supported the efficacy of VNS to treat depressive symptoms.^154^ A retrospective analysis of 25 patients with bipolar depression found similar outcomes as in patients with MDD over the course of 2 years.^155^ A case series of patients with bipolar disorder^156^ and a 1‐year prospective study of nine patients with rapid cycling bipolar disorder also provided encouraging results.^157^ The largest study to date showed that over the course of 5 years, 63% (*n* = 61/97) of patients with bipolar depression who received VNS in addition to usual care responded to treatment, compared to 39% (*n* = 23/59) of patients who received usual care. These patients also had a faster treatment response (median time to initial response: 13.7 months compared to 42.1 months, respectively), a longer period until relapse (15.2 months compared to 7.6 months) and a greater reduction in suicidality.^158^ As such, VNS could be effective in maintaining symptom remission.

#### 6.3.2 Cognition

There are no published data on the cognitive effects of VNS in bipolar disorders. However, a study of 14 patients with MDD suggested that VNS was associated with improvements across several measures of cognitive function.^159^

#### 6.3.3 Tolerability

No systematic studies have examined VNS‐related adverse events in bipolar disorders. An individual‐level meta‐analysis of more than 1000 patients with depression suggested that VNS was generally well tolerated.^160^ The most frequent adverse effects included cough, incision site pain, headaches and changes in the patient's voice. Treatment‐emergent (hypo)manic symptoms appear to be infrequent according to data from 20 bipolar depressed patients who participated in a study of depression in which manic symptoms were examined over a year.^161^ Another report also concluded that manic episodes during VNS were rare.^162^

### 6.4 Conclusion

Few studies have examined MST, DBS and VNS in bipolar disorders, highlighting the need for further research in this population. An advantage of DBS and VNS is optimal treatment adherence. Although the slow treatment effect onset associated with VNS may be undesirable in some patients, the effect of DBS occurs instantly. These protocols can be used in conjunction with most other treatments, including medication and ECT. However, due to their invasive nature, DBS and VNS remain limited to the most difficult‐to‐treat patients.

## 7 CONCLUSION

There is a growing interest and a rapidly expanding evidence base for brain stimulation as an add‐on or alternative to pharmacological and psychological treatments for bipolar disorders. For most treatment protocols, few studies were conducted exclusively in patients with bipolar disorders. However, results from many of these studies and from studies that included patients with bipolar disorders and MDD have been encouraging. Brain stimulation represents an opportunity to treat all bipolar disorder states, which usually require multiple pharmacological treatments. Treatment protocols such as deep brain stimulation or vagus nerve stimulation could overcome some of the challenges associated with the clinically complex presentation of bipolar disorders in difficult‐to‐treat patients. Novel brain stimulation protocols could address the lack of treatment options for cognitive dysfunction. Nevertheless, larger randomised controlled trials with long‐term follow‐up are needed to clarify important questions regarding treatment efficacy, tolerability, frequency of treatment‐emergent affective switches and cognitive effects (Panel 2).

PANEL 2 **Key challenges for the field and directions for future research**

**Refine treatment parameters**

Optimal treatment parameters, including electrode placement and stimulation target, may vary across bipolar disorder states, as well as within and between patients. Treatment configurations that target state‐specific brain alterations should be further investigated.

**Stratify by diagnostic subtypes**

There are limited data on how patient characteristics relate to treatment efficacy and tolerability. An important step towards stratified, and eventually personalised, treatment delivery is to examine brain stimulation within and between bipolar disorder subtypes.

**Study continuation and maintenance treatment**

Treatment is often stopped after successful mood symptom remission, despite preliminary evidence suggesting that further treatment might benefit some patients with bipolar disorders. The evidence base for continuation and maintenance treatment needs to be expanded.

**Research portable and accelerated treatment protocols**

The considerable time involved in treatment attendance and delivery remains an obstacle to the more widespread use of most brain stimulation protocols. Further research into portable devices for home use and accelerated treatment protocols is needed.

**Standardise reporting of adverse events**

It is unclear whether studies that do not report adverse effects did not collect these data or observed no adverse effects. Standardised reporting of adverse effects, including treatment‐emergent affective switches and cognitive effects, would be useful for evidence synthesis.

**Investigate experimental therapeutic protocols**

Cranial electrotherapy stimulation, focal electrically administered seizure therapy, low amplitude seizure therapy, transcutaneous vagus nerve stimulation, transcranial focused ultrasound stimulation and quadripulse stimulation lack data on patients with bipolar disorders.

## AUTHOR CONTRIBUTION

Julian Mutz conceived and wrote this review.

## FUNDING INFORMATION

JM received studentship funding from the Biotechnology and Biological Sciences Research Council (BBSRC) (ref: 2050702) and Eli Lilly and Company Limited.

## CONFLICT OF INTEREST

The funding body had no role in the writing of this review or in the decision to submit it for publication.

## ETHICAL APPROVAL

No ethical approval was needed to write this review.

## Data Availability

All data summarised in this review are available in previously published reports.
